# White matter protection with insulin-like growth factor-1 after hypoxia-ischaemia in preterm foetal sheep

**DOI:** 10.1093/braincomms/fcae373

**Published:** 2024-10-24

**Authors:** Guido Wassink, Kenta H T Cho, Sam Mathai, Christopher A Lear, Justin M Dean, Alistair J Gunn, Laura Bennet

**Affiliations:** Department of Physiology, University of Auckland, Private Bag 92019, Auckland 1023, New Zealand; Department of Physiology, University of Auckland, Private Bag 92019, Auckland 1023, New Zealand; Department of Physiology, University of Auckland, Private Bag 92019, Auckland 1023, New Zealand; Department of Physiology, University of Auckland, Private Bag 92019, Auckland 1023, New Zealand; Department of Physiology, University of Auckland, Private Bag 92019, Auckland 1023, New Zealand; Department of Physiology, University of Auckland, Private Bag 92019, Auckland 1023, New Zealand; Department of Physiology, University of Auckland, Private Bag 92019, Auckland 1023, New Zealand

**Keywords:** prematurity, hypoxia-ischaemia, insulin-like growth factor-1, white matter injury, neonate

## Abstract

Perinatal hypoxia-ischaemia in extremely preterm infants is associated with long-term neurodevelopmental impairment, for which there is no specific treatment. Insulin-like growth factor-1 can reduce acute brain injury, but its effects on chronic white matter injury after hypoxia-ischaemia are unclear. Preterm-equivalent foetal sheep (0.6 gestation) received either sham-asphyxia or asphyxia induced by umbilical cord occlusion for 30 min, and recovered for either 3 or 35 days after asphyxia. The 35 day recovery groups received either an intracerebroventricular infusion of insulin-like growth factor-1 (1 µg/24 h) or vehicle, from 3 to 14 days after asphyxia. Asphyxia was associated with ventricular enlargement, and loss of frontal and parietal white matter area (*P* < 0.05 versus sham-asphyxia). This was associated with reduced area fraction of myelin basic protein and numbers of oligodendrocyte transcription factor 2 and mature, anti-adenomatous polyposis coli-positive oligodendrocytes in periventricular white matter (*P* < 0.05), with persistent inflammation and caspase-3 activation (*P* < 0.05). Four of eight foetuses developed cystic lesions in temporal white matter. Prolonged infusion with insulin-like growth factor-1 restored frontal white matter area, improved numbers of oligodendrocyte transcription factor 2-positive and mature, anti-adenomatous polyposis coli-positive oligodendrocytes, with reduced astrogliosis and microgliosis after 35 days recovery (*P* < 0.05 versus asphyxia). One of four foetuses developed temporal cystic lesions. Functionally, insulin-like growth factor-1-treated foetuses had faster recovery of EEG power, but not spectral edge. Encouragingly, these findings show that delayed, prolonged, insulin-like growth factor-1 treatment can improve functional maturation of periventricular white matter after severe asphyxia in the very immature brain, at least in part by suppressing chronic neural inflammation.

## Teaser

Wassink and Cho *et al*. report that in extremely preterm foetal sheep central insulin-like growth factor-1 infusions starting 72 h after acute hypoxia-ischaemia and continued for 10 days improved white maturation after 35 days of recovery, at least in part by suppressing chronic neural inflammation.

## Graphical Abstract

**Graphical Abstract:**
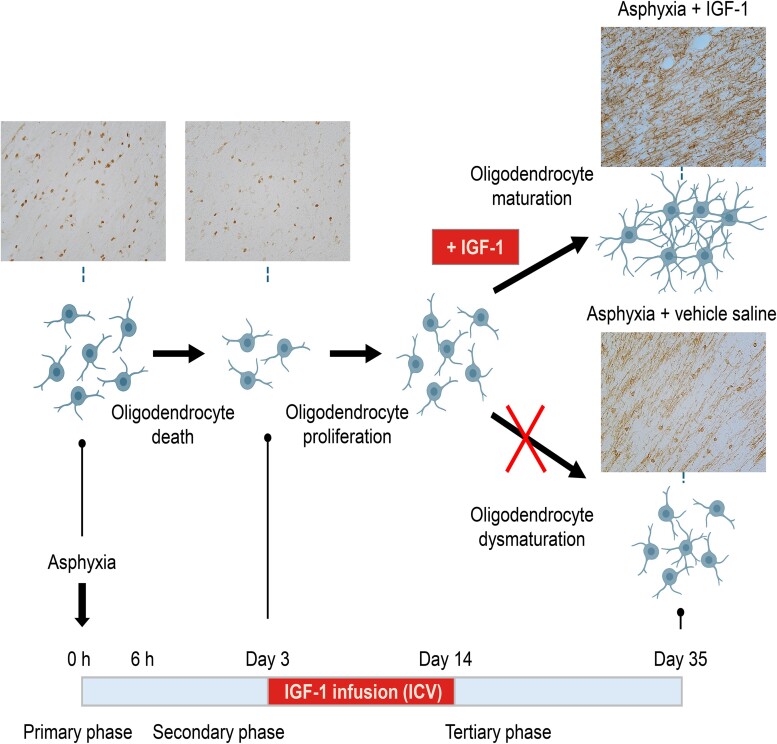

## Introduction

Extremely preterm birth remains a leading contributor to neonatal death and neurodevelopmental impairment.^1^ Most infants born before 28 weeks of gestation show white matter injury (>80%) on neuroimaging, and more than a third develop cognitive, psychiatric and neuromotor disability, including cerebral palsy.^2-4^ In part, these long-term outcomes are associated with impaired brain growth, cell maturation and neural connectivity.^5,6^ The aetiology of preterm brain injury is complex and not fully understood, and is likely multi-factorial.^7,8^ Nevertheless, perinatal hypoxia-ischaemia (HI) is a significant contributing factor and hypoxic-ischaemic encephalopathy (HIE) is more common in preterm infants than at term.^9^ Recent studies report delayed, chronic microglial activation and reactive astrogliosis, and suppressed trophic cell support after HI,^10^ which may contribute to impaired cerebral development in premature infants.^11^ For example, in preterm foetal sheep at the equivalent of 28 to 32 weeks gestation, diffuse white matter loss and microglial aggregates at 3 to 14 days after asphyxia was associated with white matter atrophy, ventriculomegaly and cystic infarction in the temporal lobe at 21 days recovery.^12^ These findings suggest a prolonged therapeutic window for white matter protection in the immature brain. Importantly, there is surprisingly little evidence in the extremely preterm brain and yet HI and neurodevelopmental disability are most common in the most preterm infants.^1,9^

The pleiotropic hormone, insulin-like growth factor-1 (IGF-1), has a critical role in foetal growth and neurodevelopment.^13^ Treatment with exogenous IGF1 can reduce mitochondrial energetic failure, apoptosis and pro-inflammation factors after neonatal HI and stroke,^14-16^ and promote glial proliferation and differentiation.^17^ Thus, it has potential to support endogenous neurorestoration and so improve long-term outcomes.^18^ In post-natal day (PD) 7 rat pups, repeated IGF-1 injections (3 mg/kg, s.c.) at 24 and 48 h after HI improved brain infarction, and cognitive and neuromotor function after 2 months.^19^ Intracerebral IGF-1 (50 µg) injections, initiated immediately after HI and repeated at 24 and 48 h post-insult also reduced striatal, hippocampal and cortical tissue loss and preserved white matter.^20,21^ In term-equivalent foetal sheep, delayed intracerebral infusion of IGF-1 (3 µg) over 1 h after cerebral ischaemia reduced oligodendrocyte loss and demyelination, and caspase-3 activation in parasagittal white matter, and increased glial proliferation.^22^ Critically, repeated injections with low-dose IGF-1 have shown greater benefit.^18^ However, intermittent therapy must be associated with marked variation in intracerebral IGF-1 concentrations. Thus, it is plausible that a sustained infusion would provide more stable levels and so be more protective.

Supporting this concept, in a phase-II randomized trial in extremely pre-term (<28 weeks gestational age, *n* = 117) infants, a continuous intravenous (i.v.) infusion of rh IGF-1/rhIGFBP-3 (250 μg/kg daily) from ≤24 h of birth until 29 weeks ± 6 days post-menstrual age was associated with an apparent trend (*P* = 0.14) towards less progression to Grades II–III germinal matrix and intraventricular, or periventricular haemorrhage, and no difference in diffuse or periventricular leukomalacia on cranial ultrasound compared with standard care.^23^ However, these imaging outcomes were a secondary endpoint of the trial. By contrast, in preterm piglets a continuous intravenous (i.v.) infusion of rhIGF-1 with binding protein (rhIGFBP)-3 (2.25 mg/kg daily) from birth to post-natal Day 19 impaired myelination in multiple brain regions and decreased hilar synapse formation, but improved balance beam performance.^24^ The reason for these differential effects is still unclear. However, interestingly, gene expression analyses indicated enhanced maturation of the GABAergic system, without affecting oligodendrocyte maturation or neuron differentiation. To date, there have been no translational studies in extreme preterm-equivalent large animals to establish whether prolonged IGF-1 therapy can protect the preterm brain after severe perinatal HI.

Thus, in the present study, we examined whether a delayed, continuous infusion of IGF-1, started 72 h after asphyxia induced by complete umbilical cord occlusion for 30 min, and continued until 14 days, would improve electrophysiological and histological outcomes at 35 days recovery in 0.6 gestation foetal sheep. In foetal sheep, this period broadly corresponds with the neural development of extremely preterm infants from 26 to 40 weeks gestation.^25^ At this gestation, oligodendrocytes are predominantly O4+ O1− preoligodendrocytes, corresponding with the highest-risk period for white-matter injury in human preterm infants.^26^

## Materials and methods

### Ethics approval

All procedures were approved by the Animal Ethics Committee (ID #N967) of The University of Auckland, under the provisions of the New Zealand Animal Welfare Act, and the Code of Ethical Conduct from the Ministry of Primary Industries, Government of New Zealand, and conforms with the ARRIVE guidelines.^27^

### Experimental preparation

Forty time-mated Romney/Suffolk cross-bred sheep were instrumented at 87 ± 2 days (d) gestation (term = 147 days), under general anaesthesia. Please see previous publications for detail of the procedures.^28,29^ Briefly, catheters and electrodes were placed for pre-ductal arterial blood sampling and to measure mean arterial blood pressure (MAP), amniotic pressure, and electrocardiographic (ECG), electroencephalographic (EEG) and nuchal electromyographic activity. A 14 mm, 22-gauge cannula and catheter was inserted into the right lateral ventricle (6 mm anterior and 4 mm lateral to bregma) for intracerebroventricular (icv) infusion with IGF-1 or vehicle, and an inflatable silicone occlude (In Vivo Metric, Healdsburg, California) placed around the umbilical cord. Twins were not instrumented, and plasma samples or tissues were not taken from these foetuses.

### Post-operative care

The protocol for post-operative care for the ewes and their foetuses has been previously reported.^28,29^

### Experimental recordings

Studies were performed at 91 ± 2 day of gestation. Foetal MAP, ECG and EEG were recorded from 24 h before the experiment to the end. The ECG signal was filtered between 0.05 and 100 Hz, digitized at 512 Hz, and used to derive foetal heart rate (FHR). The EEG signal was filtered between 1.6 and 128 Hz, and digitized at 256 Hz. EEG power and spectral edge frequency (SEF) were then extracted from the power spectrum between 1.0 and 20 Hz, using 4 s epochs. SEF was calculated as the frequency above 90% of EEG power, and presented as percentage change from baseline. EEG power was log transformed [decibels (dB), 10 × log (power)], and normalized to baseline. Physiological data were collected and analysed using Labview-based custom software (National Instruments, Auckland, New Zealand).

### Experimental protocol

Using a randomized block design, foetuses were assigned to sham asphyxia or asphyxia by total umbilical cord occlusion for 30 min, and allowed to recover for either 3 (sham 3-days, *n* = 8; or asphyxia 3-days, *n* = 8) or 35 days (sham 35-days, *n* = 8; asphyxia 35-days, *n* = 8), plus a group that received prolonged i.c.v. infusion of IGF-1 with recovery to 35 days (asphyxia 35-days-IGF-1, *n* = 8). Umbilical cord occlusion was discontinued when cardiac asystole occurred or MAP fell below 8 mmHg. Foetuses in the asphyxia-IGF-1 group received 1 µg/24 h of IGF-1 (Chiron, Emeryville, California, USA) in artificial cerebrospinal fluid (aCSF, vehicle), from 72 h until 14 days after severe asphyxia, using a microinjection pump (CMA100, Harvard Apparatus, Massachusetts, USA). The asphyxia 35-day group received the same volume of aCSF. Unfortunately, four foetal sheep in the asphyxia 35-days-IGF-1 group were required to be euthanized at varying times after recovery due to COVID-related laboratory lockdowns. Tissues and physiological data from these four animals are excluded from analyses. As well, in one case, potentially due to fixation issues, we could not achieve adequate quality for a subset of the immunohistochemical stains. Control animals were not occluded and/or received an equivalent volume of vehicle solution.

Foetal arterial blood samples were collected for measurement of pH, blood gases and glucose and lactate values 30 min before the experiment (baseline), at 5 and 25 min during occlusion, and at 1, 3, 6, 13, 20, 27 and 35 days after occlusion. Ewes and foetuses were killed with sodium pentobarbital (9 g, Pentobarb 300, Chemstock International). Foetuses were weighed, and brains fixed *in situ* with 10% formalin, processed and embedded using a paraffin preparation.

### Immunohistochemistry

Brain immunohistochemistry with diaminobenzidine staining was performed on coronal sections as per previous reports.^28,29^ In brief, labelling was performed using mouse anti-myelin basic protein (MBP; 1:200, MAB381, Merck-Millipore, Auckland, New Zealand), rabbit monoclonal anti-ionized calcium binding adapter molecule-1 (Iba1; 1:200, AB178680, Abcam), rabbit anti-glial fibrillary acidic protein (GFAP; 1:500, AB68428, Abcam), anti-cleaved cysteine-aspartic acid protease-3 (caspase-3; 1:200, 9661, Cell Signalling Technology, Danvers, Massachusetts, USA) and rabbit monoclonal anti-Ki67 (a marker of proliferation; 1:200, AB9260, Merk-Millipore). Oligodendrocytes were identified using rabbit anti-oligodendrocyte transcription factor 2 (Olig2; labels total oligodendrocytes, 1:200, AB109186, Abcam, Melbourne, Victoria, Australia) and mouse anti-adenomatous polyposis coli clone CC1 (CC1, a marker of mature oligodendrocytes; 1:50, MABC200, Merk-Millipore).

### Immunofluorescence

Immunofluorescence was performed as previously reported.^30^ In brief, to assess the type of proliferating cells, slides were incubated overnight with 1:50 rabbit anti-Ki-67 (AB9260, Merk-Millipore) and 1:50 mouse anti-CC1 (MABC200, Merk-Millipore). To evaluate cells undergoing apoptosis, tissue sections were incubated with 1:200 anti-cleaved caspase-3 (9661, Cell Signalling Technology, Danvers, Massachusetts, USA) and 1:50 mouse anti-CC1 (MABC200, Merk-Millipore). Counter-staining to identify nuclei was performed using 4′,6-diamidino-2-phenylindole (1:10,000, D1306, ThermoFisher, Victoria, Australia), with negative controls run in parallel.

### White matter area measurements

**Figure 1:**
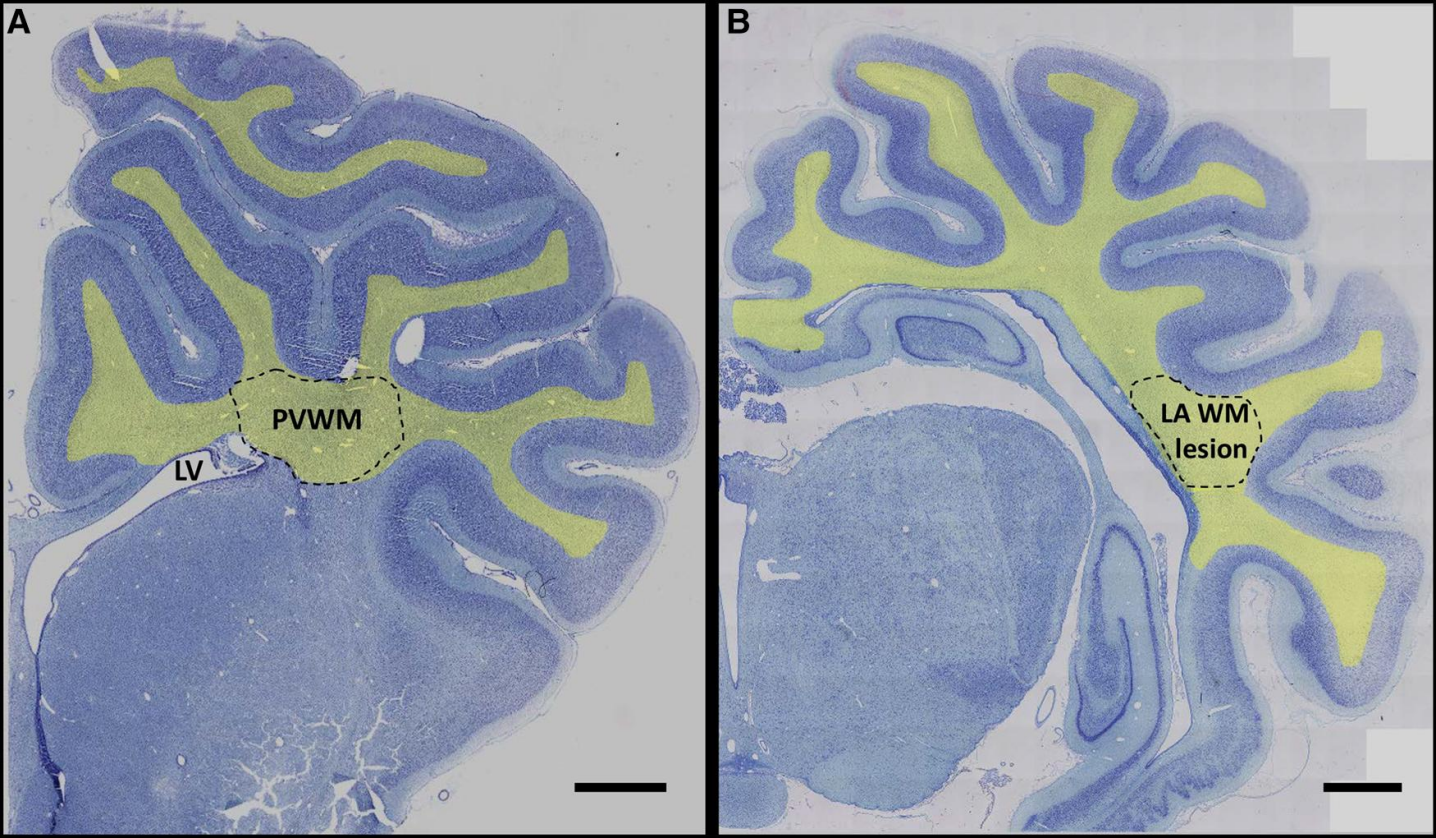
**Photomicrographs showing anatomical fields used for histological analysis.** Area measurements (mm^2^) of the lateral ventricle and total white matter were obtained at 21 mm (panel **A**) and 17 mm (panel **B**) anterior to stereotactic zero. Total white matter areas, shown by shading, included all white matter of the frontal and temporal lobes (21 mm), and parietal and temporal lobes (17 mm), of both hemispheres. Olig2-, MBP-, Iba1-, GFAP-, caspase3- and Ki67-positive cells were quantified in periventricular white matter (panel **A**). Infarct area was determined in the temporal lobe (panel **B**), as demarcated by the dotted lines. LV, lateral vehicle; PVWM, periventricular white matter; TAWM, temporal white matter. Scale bar = 2 mm.

Macroscopic assessment of the lateral ventricles and total white matter at the level of the mid-striatum and dorsal hippocampus was conducted on acid-fuchsin/thionine sections (Fig. 1), using a Zeiss Axio Imager Z2 microscope (Carl Zeiss, Auckland, New Zealand) equipped with Vslide (Metasystems, Altlussheim, Germany). Area (mm^2^) measurements were obtained at 10 × magnification (0.45NA, Plan Apochromatic) by tracing around the ventricles, and white matter tracts. Infarction in the lateral white matter at the level of the hippocampus was assessed on Iba1-stained sections, and defined as regions with gross cell loss and disintegrated tissue architecture. One tissue section per animal was used, and data from left and right hemispheres were averaged.

### Immunohistological quantification

Immunohistological changes were quantified on sections taken at 21 mm anterior to stereotaxic zero. Digital images were obtained from MBP and CC1-stained sections at 20 × magnification on a Nikon Eclipse 80i microscope with NIS-Elements 5.0 software (Coherent Scientific, Thebartin, South Australia, Australia) using one field in the periventricular white matter. Area fraction for MBP-labelled slides was then calculated with ImageJ software using the default threshold (National Institute of Health). Cell counts of mature oligodendrocytes (CC1-positive) were performed manually using Image J software (National Institute of Health). Using StereoInvestigator software (MBF Bioscience, Williston, Vermont, USA), the total white matter of each hemisphere were traced at 2 × magnification, and immuno-positive cells (Olig2, Iba1, GFAP, caspase3 and Ki67) quantified with a fractionator probe (20 × objective, sampling grid: 600 μm × 600 μm, counting frame: 100 µm × 100 µm). Cells touching the top and left hand boundaries were excluded. Cleaved caspase-3 positive cells that exhibited staining and apoptotic bodies, and amoeboid and ramified microglia were counted.

### Statistical analysis

**Figure 2:**
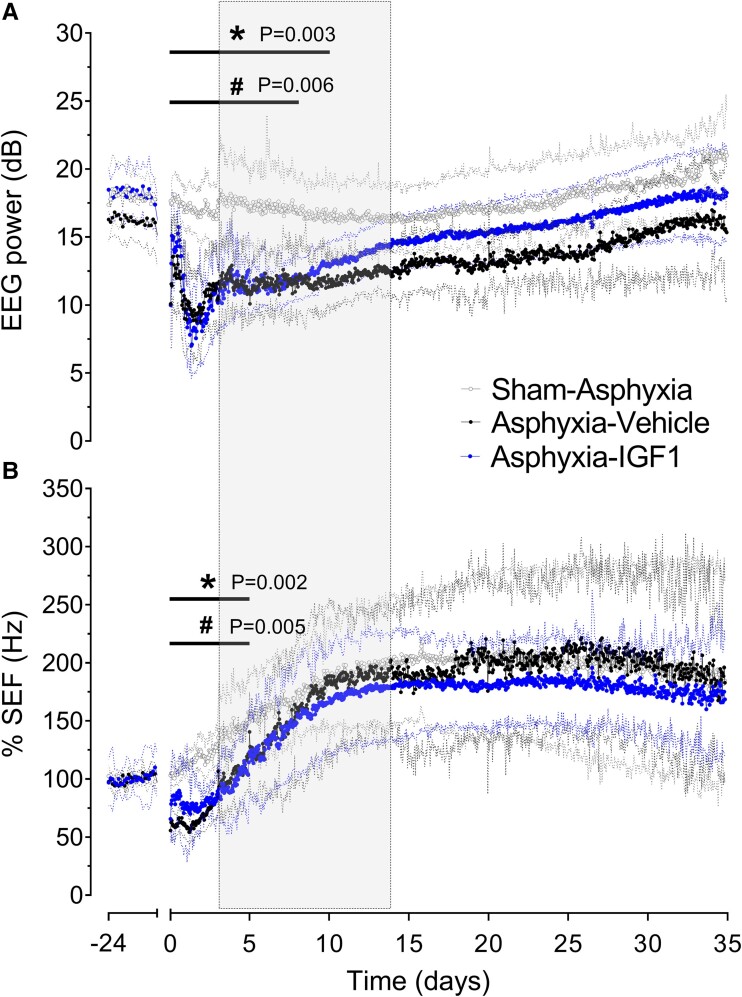
**Time sequence of foetal electrophysiological changes.** Foetal EEG power (dB; panel **A**) and spectral edge frequency (%SEF; Hz; panel **B**) from 24 h before until 35 days after 30 min of umbilical cord occlusion in the sham 35-days (open circles; *n* = 8), asphyxia 35-days (closed circles; *n* = 8) and asphyxia 35-days-IGF-1 groups (blue circles; *n* = 4). The period of umbilical cord occlusion is omitted. IGF-1 or vehicle infusion from 3 to 14 days post-asphyxia is denoted by the shaded area. Note that EEG power, but not SEF, recovered faster in animals that received IGF-1. Data are presented as mean ± 95% CI; hourly averages. Group comparisons by repeated-measures 2-Factor ANOVA, and LSD *post hoc* test. *P < 0.05, sham 35-days versus asphyxia 35-days; ^#^*P* < 0.05, sham 35-days versus asphyxia 35-days-IGF-1. dB, decibel; Hz, hertz; IGF-1, insulin-like growth factor-1; SEF, spectral edge frequency.

All data and histological analyses were blinded through coding of treatment groups. There were no significant differences in blood composition or physiological data between 3-day and 35-day animals either before, during or after (sham) asphyxia, and hence these group data were merged in Table 1 and Fig. 2. Signal artefact precluded EEG analysis in two asphyxia foetuses. The effects of asphyxia and IGF-1 on biochemical and physiological parameters were assessed using a mixed-model ANOVA, with time as a repeated measure. Baseline values were included as covariate where applicable (SPSS Statistics, IBM, Armonk, New York, USA). Between-group comparisons of histological data and area measurements were performed by unpaired *t*-test or one-way ANOVA followed by Fisher’s least-significant difference (LSD) test when an overall group or interaction effect was found. Biometric data were compared by unpaired *t*-test. Incidences and binary data were tested with the Fisher Exact test. Data are presented as mean ± standard deviation (SD) or 95% confidence interval (CI). Data were considered significant at *P* < 0.05.

**Table 1:** Foetal blood composition parameters

Variable	Group	Baseline	5 min occlusion	25 min occlusion	+1 day	+3 days	+6 days	+13 days	+20 days	+27 days	+35 days
pH	Sham	7.39 ± 0.02	7.38 ± 0.02	7.39 ± 0.02	7.37 ± 0.02	7.36 ± 0.02	7.38 ± 0.02	7.38 ± 0.02	7.39 ± 0.02	7.38 ± 0.01	7.38 ± 0.02
	Asphyxia	7.39 ± 0.02	7.05 ± 0.09^a^	6.79 ± 0.14^a^	7.38 ± 0.02	7.37 ± 0.03	7.37 ± 0.03	7.36 ± 0.03	7.35 ± 0.03	7.37 ± 0.03	7.35 ± 0.06
	Asphyxia-IGF-1	7.38 ± 0.01	7.09 ± 0.01	6.74 ± 0.03	7.39 ± 0.02	7.39 ± 0.02	7.38 ± 0.01	7.38 ± 0.02	7.39 ± 0.02	7.37 ± 0.02	7.36 ± 0.04
PaCO_2_ (mmHg)	Sham	47.2 ± 3.4	44.6 ± 3.7	48.0 ± 3.7	49.0 ± 3.6	48.3 ± 2.2	48.6 ± 3.8	48.7 ± 2.0	49.6 ± 4.3	48.4 ± 4.3	48.7 ± 3.9
	Asphyxia	46.3 ± 2.8	95.3 ± 24.0^a^	153.7 ± 13.8^a^	46.6 ± 4.0	46.4 ± 3.2	45.5 ± 2.8	47.8 ± 3.1	49.8 ± 3.4	47.1 ± 2.0	49.8 ± 3.8
	Asphyxia-IGF-1	44.6 ± 3.0	92.2 ± 2.6	175.2 ± 5.6^b^	42.8 ± 1.3	45.1 ± 10.4	46.8 ± 1.8	44.3 ± 2.6	45.3 ± 4.1	47.9 ± 2.7	51.0 ± 6.7
PaO_2_ (mmHg)	Sham	22.9 ± 2.0	23.1 ± 1.8	22.7 ± 1.7	22.3 ± 1.8	22.7 ± 2.3	22.4 ± 2.5	23.3 ± 1.8	21.2 ± 1.9	21.1 ± 4.9	20.7 ± 3.0
	Asphyxia	23.9 ± 3.0	6.9 ± 3.3^a^	10.6 ± 4.1^a^	24.9 ± 2.7^a^	25.1 ± 2.5^a^	27.3 ± 3.9	27.2 ± 3.9	26.2 ± 4.4	26.2 ± 4.4	24.2 ± 4.8
	Asphyxia-IGF-1	27.0 ± 1.9^c^	8.9 ± 1.2	19.5 ± 4.2^b^	31.1 ± 2.2^b^	30.3 ± 2.0^b^	30.3 ± 2.0	31.3 ± 2.9	31.5 ± 2.0	26.0 ± 2.0	23.2 ± 7.7
Glucose (mmol/l)	Sham	1.0 ± 0.2	1.0 ± 0.2	1.1 ± 0.2	1.1 ± 0.2	1.1 ± 0.2	0.9 ± 0.2	0.8 ± 0.1	0.7 ± 0.2	0.7 ± 0.3	0.7 ± 0.2
	Asphyxia	1.2 ± 0.3	0.5 ± 0.4^a^	0.5 ± 0.5^a^	1.2 ± 0.2	1.2 ± 0.2	1.0 ± 0.2	1.0 ± 0.2	1.0 ± 0.2	1.0 ± 0.2	0.9 ± 0.3
	Asphyxia-IGF-1	1.2 ± 0.1	0.4 ± 0.1	0.6 ± 0.4	1.3 ± 0.1	1.4 ± 0.1	1.1 ± 0.0	1.1 ± 0.1	1.2 ± 0.2	1.2 ± 0.1	1.0 ± 0.3
Lactate (mmol/l)	Sham	0.9 ± 0.2	0.9 ± 0.4	0.9 ± 0.3	1.0 ± 0.4	0.9 ± 0.2	0.7 ± 0.1	0.7 ± 0.1	0.7 ± 0.1	0.7 ± 0.1	0.8 ± 0.1
	Asphyxia	0.8 ± 0.2	4.1 ± 1.4^a^	7.9 ± 0.7^a^	0.9 ± 0.2	1.0 ± 0.4	0.7 ± 0.2	0.7 ± 0.2	0.7 ± 0.1	0.7 ± 0.0	0.8 ± 0.3
	Asphyxia-IGF-1	0.7 ± 0.2	3.5 ± 0.3	7.6 ± 0.5	0.8 ± 0.2	0.9 ± 0.2	0.9 ± 0.2	0.8 ± 0.3	0.9 ± 0.3	0.9 ± 0.2	0.9 ± 0.2

Foetal arterial blood samples were taken from sham 3-days and 35-days, asphyxia-vehicle 3-days and 35-days, and asphyxia-IGF-1 35-days foetuses at −60 min before cord occlusion (baseline), at 5 and 25 min during cord occlusion, and at 1, 3, 6, 13, 20, 27 and 35 days after occlusion. Values of the 3 and 35 days recovery animals were averaged. PaCO_2_, arterial pressure of carbon dioxide; PaO_2_, arterial pressure of oxygen; IGF-1, insulin-like growth factor-1. Data are presented as means ± SD. Between group comparisons by repeated measures ANOVA and one-way ANOVA with LSD *post hoc* test.

^a^ *P* < 0.001 versus sham asphyxia.

^b^ *P* < 0.001 versus asphyxia-vehicle.

^c^ *P* = 0.03 versus Sham.

Based on a pooled SD of 178 for Olig2-positive oligodendrocyte survival in parasagittal white matter after asphyxia, a group size of *n* = 5 would provide 80% power (*α* = 0.05) to detect a difference of ≥400 cells per field of view between groups.

## Results

### Blood composition and physiology before and during asphyxia

There were no significant baseline differences between groups for EEG, SEF, FHR pH, PaCO_2_ or glucose or lactate values (one way ANOVA; n.s.). Foetal baseline MAP and PaO_2_ were higher in asphyxia 35-day-IGF-1 animals than in asphyxia 35-day animals (one-way ANOVA, MAP: *F*(2, 16) = 4.0, *P* = 0.02; PaO_2_: *F*(2, 32) = 4.4, *P* = 0.03). Umbilical cord occlusion led to considerable reduction in pH and PaO_2_, and increase in PaCO_2_ and lactate (Table 1), with immediate suppression of EEG and SEF (Fig. 2), and rapid onset of bradycardia and hypertension, followed by progressive development of near-terminal hypotension (Supplementary Fig. S1). Intra-occlusion FHR and MAP responses were not different between groups (repeated measures ANOVA; n.s.), but IGF-1 treated animals had a higher PaCO_2_ and PaO_2_ at 25 min of occlusion (versus asphyxia, PaCO_2_: *F*(2, 29) = 592.6, *P* < 0.001; PaO_2_: *F*(2, 29) = 53.2, *P* < 0.001).

Two animals in the asphyxia 35-day group had their occlusion released at 28 and 29 min due to intermittent cardiac asystole. The total duration of asphyxia (time; 29.3 ± 0.5 min), depth of EEG and SEF suppression (nadir; 0.5 ± 4.0 dB and 2.3 ± 0.5 Hz) and FHR and MAP nadir (end of occlusion; 59.7 ± 5.2 bpm and 14.0 ± 1.4 mmHg) were not different between groups (one-way ANOVA; n.s.). Sham-asphyxia was not associated with changes in blood composition (Table 1), EEG (18.0 ± 3.0 dB) or SEF (6.7 ± 1.7 Hz) (Fig. 2), or FHR (193.7 ± 8.0 bpm) or MAP values compared to baseline (35.5 ± 3.6 mmHg, Supplementary Fig. S1).

### Blood composition and physiological changes after asphyxia

After umbilical cord occlusion was released, rebound tachycardia and hypertension returned to baseline within 30 min (Supplementary Fig. S1), and pH, PaCO_2_ and glucose and lactate values recovered to sham-asphyxia levels within 24 h (Table 1). The asphyxia and asphyxia 35 day-IGF-1 group had increased PaO_2_ levels over sham-asphyxia at days 1 and 3 post-occlusion (Table 1), with greater PaO_2_ in IGF-1 treated animals than asphyxia-vehicle (asphyxia 35-days-IGF1 versus asphyxia 35-days; one-way ANOVA, Day 1: *F*(2, 31) = 24.7, *P* < 0.003; Day 3: *F*(2, 30) = 16.7, *P* < 0.01).

### EEG power, SEF after asphyxia, and post-mortem data

In the asphyxia 35-days group, EEG power and SEF remained suppressed until 10 and 5 days after cord occlusion (versus shams, repeated measures ANOVA, EEG power: *P* = 0.003; SEF: *P* = 0.002; Fig. 2), respectively. Asphyxia 35-days-IGF-1 was associated with faster recovery of EEG power than asphyxia 35-days, but not SEF, such that EEG power returned to sham values at Day 8 post-occlusion. At post-mortem, there were no significant differences in the proportion of foetal sex, or singleton to multiple pregnancies (Fisher’s Exact Test; n.s., Table 2), or foetal core or liver weight between groups at either 3 or 35 days (unpaired *t*-test; n.s., Table 2) post-occlusion. Foetal brain weight was reduced at 3 days after asphyxia (versus shams, 18.6 ± 0.6 versus 21.3 ± 0.6 g; unpaired *t*-test, *P* = 0.01). By contrast, there was no difference between the sham 35-days, asphyxia 35-days or asphyxia 35-days-IGF-1 groups at post-mortem (unpaired *t*-test; 43.5 ± 4.7 versus 41.1 ± 2.9 versus 39.1 ± 2.1 g; n.s. Table 2).

**Table 2:** Foetal biometric parameters

Experimental group	*N*	Body weight (kg)	Brain weight (g)	Liver (g)	Singleton:twins	Female:male
Sham 3-days	8	0.9 ± 0.1	21.3 ± 0.6	65.7 ± 11.7	7:1	5:3
Asphyxia 3-days	8	1.1 ± 0.0	18.6 ± 0.6^a^	63.3 ± 8.0	8:0	5:3
Sham 35-days	8	2.8 ± 0.3	43.5 ± 4.7	92.3 ± 23.5	7:1	3:5
Asphyxia 35-days	8	3.0 ± 0.3	41.1 ± 2.9	96.4 ± 30.9	7:1	4:4
Asphyxia 35-days-IGF-1	4	3.0 ± 0.4	39.1 ± 2.1	120.6 ± 28.0	4:0	1:3

Note that only one foetus was instrumented per ewe and second twins were not instrumented. Data presented as means ± SD. Group comparisons by unpaired *t*-test.

IGF-1, insulin-like growth factor-1.

^a^ *P* = 0.01 versus sham 3-days.

### White matter and ventricular area, and temporal cystic infarcts

**Figure 3:**
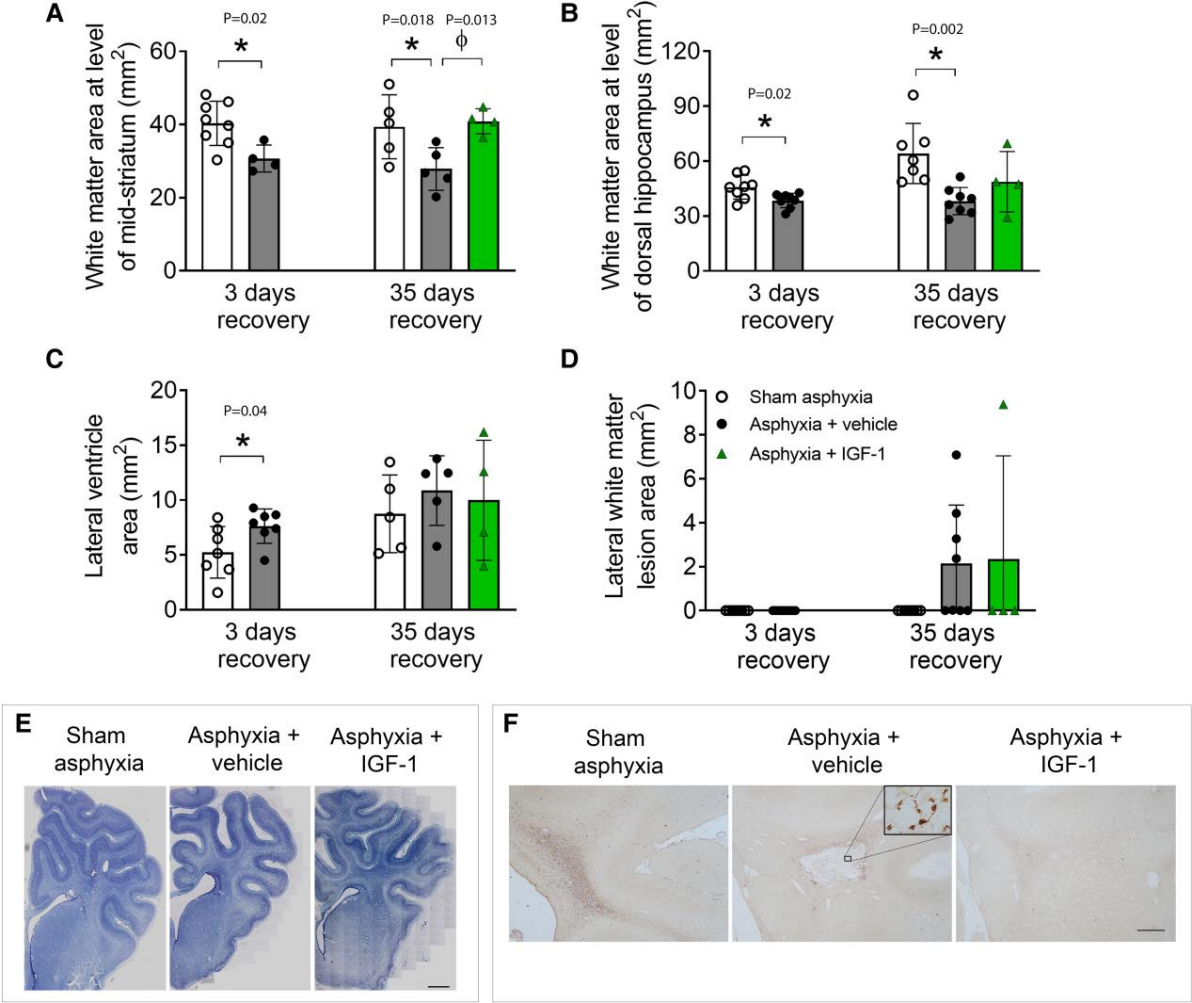
**Area measurements and cystic infarcts.** Total area of frontal (panel **A**) and parietal white matter (panel **B**), the lateral ventricle (panel **C**) and cystic infarctions (panel **D**) in the temporal white matter in mm^2^ in sham (sham-asphyxia 3- and 35-days, white bars and circles), asphyxia-vehicle (asphyxia 3- and 35-days, black bars and circles) and IGF-1 infusion (asphyxia 35-days-IGF-1, green bars and triangles) animals. Note the ventricular dilatation at 3, but not 35 days recovery, and sustained reduction in frontal and parietal white matter area after global asphyxia, which was prevented with IGF-1 treatment in frontal, but not parietal white matter. The incidence of macroscopic infarction was not different between asphyxia-vehicle and IGF-1 infusion foetuses (Fisher Exact test, *P* = 0.41). Bar data presented as mean ± SD; symbols represent animals in each group. Group comparisons by unpaired *t*-test for groups at 3-day recovery, and ANOVA with LSD *post hoc* testing for groups at 35-day recovery. *P < 0.05 versus sham asphyxia; ϕ*P* < 0.05 versus asphyxia-vehicle. Photomicrographic examples of ventriculomegaly at 3 days after asphyxia (1 × magnification, panel **E**), and cystic infarction in lateral white matter at 35 days after asphyxia (scale bar = 2 mm, panel **F**). IGF-1, insulin-like growth factor-1.

Asphyxia was associated with reduced total white matter area at the level of the mid-striatum (*t*-test and one-way ANOVA at 3 and 35 days; 3 days: *P* = 0.02; 35 days: *F*(2, 15) = 5.6, *P* = 0.018; Fig. 3A) and hippocampus (3 days: *P* = 0.02; 35 days: *F*(2, 16) = 7.2, *P* = 0.002; Fig. 3B). Prolonged IGF-1 treatment restored striatal, but not hippocampal, white matter area to sham levels (versus asphyxia 35-days, 40.9 ± 3.4 versus 27.9 ± 5.8 mm^2^; one-way ANOVA, *F*(2, 15) = 5.6, *P* = 0.013, Fig. 3A and B). Asphyxia was associated with enlargement of the lateral ventricle at 3 days post-occlusion (versus sham-asphyxia; 7.6 ± 1.1 versus 5.2 ± 2.4 mm^2^; unpaired *t*-test, *P* = 0.04), but not at 35 days post-occlusion (one-way ANOVA, n.s). Ventricular area after IGF-1 infusion was not different from sham-asphyxia or asphyxia-vehicle at 35 days (one-way ANOVA, n.s., Fig. 3C and E). There were no foetuses with macroscopic infarcts in lateral white matter at 3 days after asphyxia-vehicle or sham-asphyxia. Four of eight asphyxia-vehicle foetuses and one of four asphyxia-IGF-1 foetuses developed infarcts in lateral white matter at 35 days post-occlusion (Fisher exact, *P* = 0.58, Fig. 3D and F).

### Immunohistological outcomes

**Figure 4:**
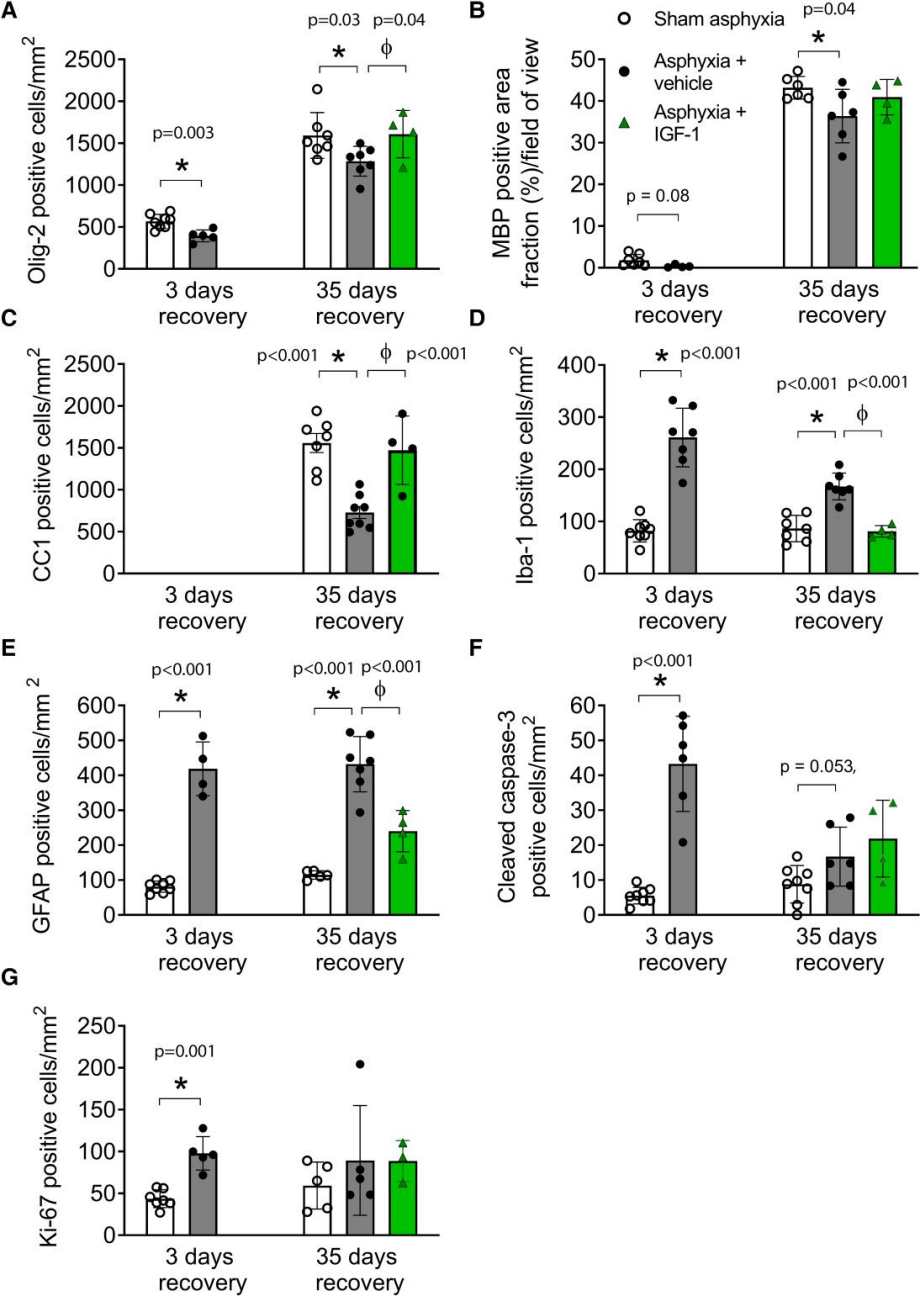
**Histological quantification.** Changes in numbers (cells/mm^2^) and area fraction (%/field of view) of total oligodendrocytes (Olig2, panel **A**), myelin (MBP, panel **B**), mature oligodendrocytes (CC1, panel **C**), microglia (Iba1, panel **D**), astrocytes (GFAP, panel **E**), apoptotic cells (cleaved caspase-3, panel **F**) and proliferating cells (Ki67, panel **G**), in the periventricular white matter at 3 and 35 days after sham asphyxia (white bars and circles), asphyxia + vehicle (black bars and circles) or asphyxia + IGF-1 infusion (green bars and triangles). Sham 3-days (*n* = 8), asphyxia-vehicle 3-days (*n* = 8), sham 35-days (*n* = 8), asphyxia 35-days (*n* = 8) and asphyxia 35-days-IGF-1 (*n* = 4) groups. Bar data are mean ± SD; symbols represent animals in each group. Between-group comparisons by unpaired *t*-test for groups at 3-days recovery, and ANOVA with LSD *post hoc* for groups at 35-days recovery. *P < 0.05 versus sham asphyxia, ϕ*P* < 0.05 versus asphyxia. IGF-1, insulin-like growth factor-1.

**Figure 5:**
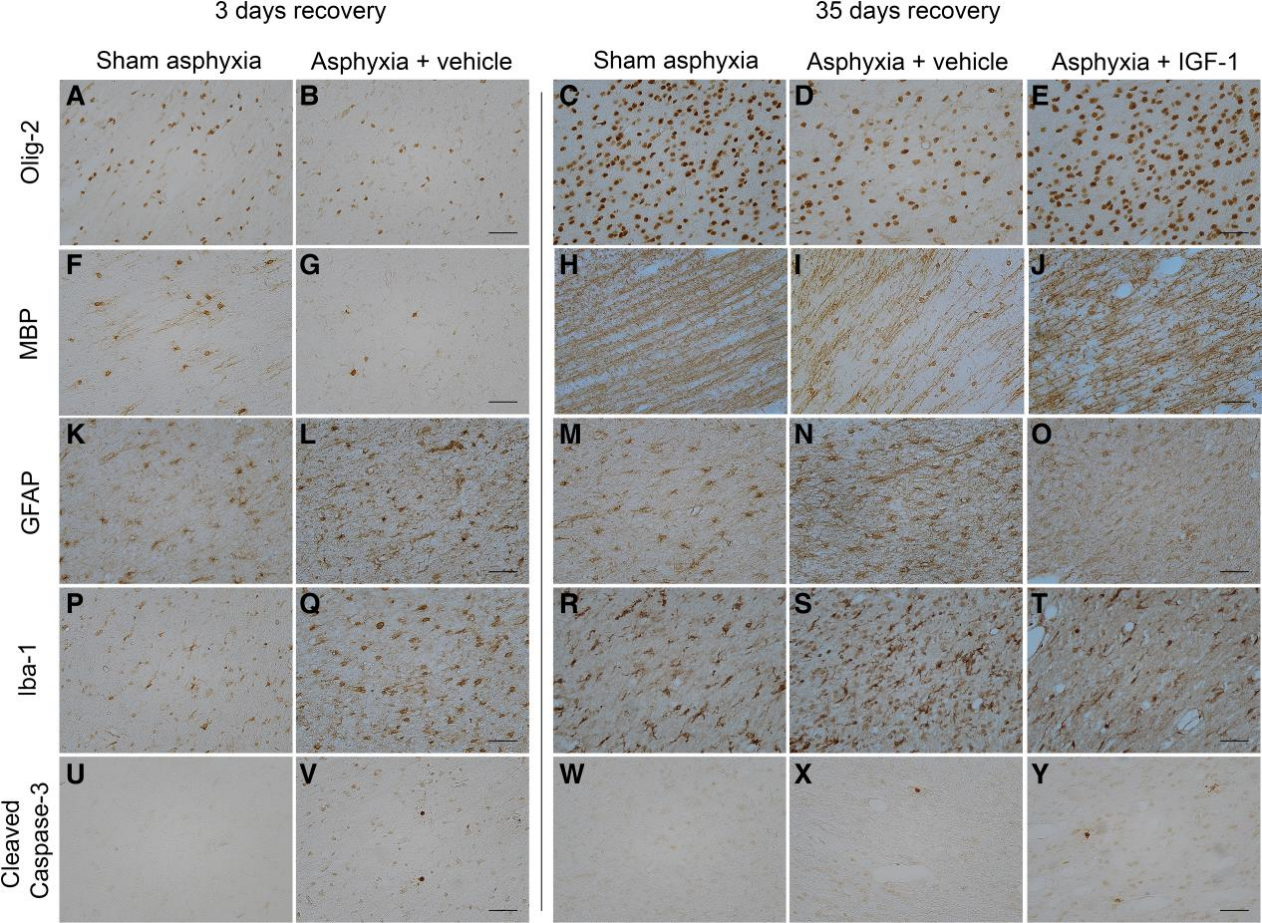
**Photomicrographic examples.** Total oligodendrocytes (Olig-2; panels **A–E**), myelin basic protein (MBP; panels **F–J**), astroglia (GFAP; panels **K–O**), microglia (Iba1; **panels P–T)** and apoptotic cells (cleaved caspase-3; panels **U–Y**) in periventricular white matter of sham 3-days, asphyxia 3-days, sham 35-days, asphyxia 35-days and asphyxia 35-days-IGF-1 animals. Scale bar = 50 µm. IGF-1, insulin-like growth factor-1.

Asphyxia was associated with prolonged loss of total, Olig2-positive oligodendrocytes in periventricular white matter at 3 and 35 days after occlusion (versus shams, *t*-test and one-way ANOVA; 3 days: *P* = 0.003; 35 days: *F*(2, 15) = 3.6, *P* = 0.03; Figs. 4A and 5), and reduced MBP-positive mature myelin (*t*-test and one-way ANOVA; 3 days: *P* = 0.080; 35 days: *F*(2, 16) = 11.6, *P* = 0.04; Figs. 4B and 5). Similarly, there was marked loss of mature CC1-positive oligodendrocytes at 35 days after asphyxia (one-way ANOVA, *F*(2, 16) = 17.99, *P* < 0.001) compared to shams (Fig. 4C and Supplementary Fig. S2). IGF-1 infusion restored both total Olig2 and mature CC1 oligodendrocytes at Day 35 to sham levels (one-way ANOVA, Olig2: *F*(2, 15) = 3.6, *P* = 0.04; CC1: *F*(2, 16) = 17.99, *P* < 0.001, Figs. 4A–C and 5). Note that mature CC1-positive oligodendrocytes were not seen in any animals 3 days after occlusion (data not shown).

Asphyxia was associated with greater numbers of Iba1-positive microglial cells in periventricular white matter (versus shams; *t*-test and one-way ANOVA at 3 and 35 days, 3 days: *P* < 0.001; 35 days: *F*(2, 15) = 27.0, *P* < 0.001; Figs. 4D and 5) and GFAP-positive astrocytes compared with shams (*t*-test and one-way ANOVA at 3 and 35 days; 3 days: *P* < 0.001; 35 days: *F*(2, 13) = 40.6, *P* < 0.001, Figs. 4E and 5). IGF-1 infusion was associated with partial reduction in numbers of microglia and astroglia to sham levels at 35 days of recovery (versus asphyxia, one-way ANOVA; microglia: *F*(2, 15) = 27.0, *P* < 0.001; astroglia: *F*(2, 13) = 40.6, *P* < 0.001; Figs. 4D and E and 5).

Finally, asphyxia was associated with increased numbers of cleaved caspase3-positive cells in periventricular white matter at 3 and 35 days recovery (versus shams; *t*-test and one-way ANOVA, 3 days: *P* < 0.001; 35 days: *F*(2, 15) = 4.1, *P* = 0.053; Figs. 4F and 5), whereas Ki67-positive cells were increased at 3 days (versus shams; unpaired *t*-test, *P* = 0.001) but not at 35 days after asphyxia (one-way ANOVA, n.s., Figs. 4F and G and 5). Asphyxia 35-days-IGF-1 had no significant effect on caspase-3 positive cells or Ki67 proliferating cells compared to asphyxia 35-days (one-way ANOVA, n.s., Fig. 4F and G). In double-labelling, Ki67-positive proliferating cells, but not apoptotic caspase-3 cells, co-expressed with mature CC-1 oligodendrocytes (Supplementary Fig. S3).

## Discussion

White matter injuries in survivors of preterm birth include diffuse and necrotic cystic elements, which in turn are highly associated with subsequent impaired neurodevelopment and cerebral palsy.^31-33^ In modern post-mortem cohorts, persistent astrocytosis and microglial infiltration after preterm birth are associated with demyelination, and reduced regional and global cerebral volumes, and cortical complexity.^10,34^ At present, there are no established interventions that protect or repair white matter in high-risk preterm infants. In the current study, we show for the first time in preterm foetal sheep that a delayed, prolonged i.c.v. infusion of IGF-1 started at three days and continued until 14 days after severe, global asphyxia, restored numbers of total (Olig2-positive) and mature CC1-positive oligodendrocytes and MBP-positive myelin in periventricular white matter, with suppression of astroglial and microglial activation, but not residual apoptosis, after 35 days recovery. IGF-1 preserved frontal, but not parietal, white matter, and had no significant effect on ventricular enlargement or the incidence of temporal infarction. Functionally, these histologic improvements were associated with faster recovery of EEG power. These data suggest that even very delayed IGF-1 therapy after perinatal HI may have potential to improve white matter maturation in preterm infants with HIE.

The current paradigm of prolonged, global asphyxia, induced by complete umbilical cord occlusion, led to preterm cerebral injury defined by ventriculomegaly and white matter atrophy in the forebrain, with sustained hypomyelination, glial inflammation and cell death, consistent with imaging and necroptic studies in preterm infants with HIE.^9,34,35^ It is now well established that neuronal and white matter injury evolve after HI.^36^ After reperfusion after acute asphyxia, cell oxidative function is transiently restored for ∼6–8 h, followed by progressive metabolic failure, delayed seizures and cell death.^37^ Interestingly, in the present study foetal oxygen levels were transiently increased for 3 days after asphyxia, likely reflecting a combination of reduced body movements and reduced cerebral oxygen consumption.^38^

In practice, the treatment window for most neuroprotective strategies, including therapeutic hypothermia, is the relatively brief latent phase.^36,39^ Hypothermia is not recommended for preterm infants due to greater risk of death and side-effects,^40^ although in preclinical studies, cooling in preterm-equivalent foetal sheep in this interval was neuroprotective.^41,42^ Consistent with this, in near-term foetal sheep a low-dose IGF-1 (3 µg) icv infusion initiated at 90 min after global cerebral ischaemia and continued for either 1 h or 24 h, reduced oligodendrocyte and myelin loss, and caspase-3 induction in parasagittal white matter after 4 days recovery.^22,43^ It is important to consider that in clinical practice starting therapeutic treatment shortly after birth is logistically challenging, and neuroprotection is largely lost with greater treatment delay.^44^

The present study shows, strikingly, that a prolonged IGF-1 infusion delayed until 72 h after asphyxia, well after the latent phase, at a time when secondary seizures have resolved, restored total numbers of oligodendrocytes and frontal white matter myelination. This suggests that this very delayed treatment targeted alternative mechanisms.^45^ This wide potential therapeutic window is important, since the precise timing of the preterm cerebral injury is often unclear,^36^ and injury may be triggered by chronic or repeated insults before birth.^46^ Consistent with the current results, in foetal sheep studies at 0.7 gestation, acute asphyxia triggered marked pre-oligodendrocyte and myelin loss by 3 days.^41,42^ In several studies, although active cell proliferation restored total number of oligodendrocytes to sham control values by 7 days recovery, there was a marked reduction in pre-myelinating oligodendrocytes and white matter volume after 3 weeks, suggesting that oligodendrocyte precursors failed to mature into myelin producing cells.^12,29^ Supporting this, corpus collosum thinning, periventricular leucomalacia, delayed myelination and cortical atrophy are prevalent injuries at mid-childhood in ex-preterm infants who had been exposed to acute hypoxaemia and metabolic acidosis at birth.^47,48^

In the current cohort, restoration of both total (Olig2-positive) and mature (CC1-positive) oligodendrocytes to sham-control levels after IGF-1 treatment after 5 weeks recovery was associated with partial improvement in MBP myelin, suggesting greater oligodendrocyte survival and improved maturation but some persistent impairment of oligodendrocyte function. Further, intact white matter area was preserved in the subcortical forebrain, but not temporal subcortex more distal from the lateral ventricles. In adult rats with HI cerebral injury, after intraventricular administration, IGF-1 (3 µg) diffused within 1 h to distal neuronal and glial cells via the perivascular circulation and ependyma-white matter tract route.^49,50^ However, ependymal development is not complete until 34 weeks of gestation in humans.^51^ Thus, speculatively, in the present study, immaturity of the ependyma may have limited the amount of IGF-1 reaching the temporal lobe.^52^ Further, IGF-1 protein has a short half-life of less than ∼5 min, and crosses the blood brain barrier via saturable transport mechanisms, which will have limited bioavailability.^53^

Consistent with its insulin like-effects, in children with post-natal growth failure treatment with rhIGF-1 was associated with hypoglycaemia.^54^ By contrast, in the present study the prolonged i.c.v. IGF-1 infusion was not associated with reduced blood sugar levels or differences in organ weights, suggesting mostly local effects.^55^ Alternatively, intranasal administration has been suggested as is a non-invasive, effective treatment route to enable absorption in to brain and spinal fluid.^56^ The barrier between the cribriform foramina and parenchyma is minimal, allowing large molecule transport towards injured tissues.^57^ This will be an important focus of future research.

The precise mechanisms that underlie dysmaturation of oligodendrocyte function after HI include microglial activation, abnormalities of astroglial-produced hyaluronic acid in the extracellular matrix, and impaired oligodendroglial-axonal signalling.^58-60^ For example, in preterm foetal sheep that received HI, diffuse white matter injury was associated with gliotic lesions in which arrested pre-oligodendrocyte differentiation was directly proportional to the magnitude of astrogliosis.^34,61^ In the present study, the finding that MBP-myelin restoration was partial, despite suppression of Iba1-labeled microgliosis to sham-control values, supports the hypothesis that persistent astrogliosis is a significant mediator of oligodendrocyte dysmaturation and long-term myelination failure.^62^ However, pro-inflammatory microglia promote formation of reactive astrocytes.^63^ Thus, speculatively, microglial suppression by IGF-1 may have indirectly contributed to improved oligodendrocyte maturation and myelination. Importantly, these data extend our previous studies at a later stage of maturation (0.7 gestation, broadly equivalent to ∼30–32 weeks gestation in humans), in which late anti-inflammatory treatment with either human amnion epithelial cells or etanercept, a TNF inhibitor, restored mature oligodendroglia in the parietal and temporal white matter at 3 weeks after asphyxia.^29,46^

IGF-1 is an important endogenous pro-survival hormone,^11^ that plays a key role in maturation of oligodendrocytes.^64^ There is conflicting evidence on the impact of IGF-1 on neural inflammation after LPS infection or HI.^15^ For example, intranasal IGF-1 attenuated microglial activation and invasion of polymorphonuclear leukocytes in rat parenchyma after LPS exposure,^65^ while intracerebral IGF-1 reduced cytokines in juvenile rats with forebrain stroke, including interleukin (IL) -6, IL-10 and TNF.^66^ In near-term foetal sheep, i.c.v. infusion of IGF-1 prevented loss of oligodendrocytes and myelin density at four days after cerebral ischaemia, in association with reduced caspase-3 induction but enhanced microglial proliferation.^67^ Interestingly, greater oligodendrocyte numbers were due to depressed apoptosis and increased proliferation, whereas reactive microcytosis and astrogliosis were related to greater proliferation alone,^22^ consistent with previous reports in neonatal rats.^57,68^

In the current study, low-levels of apoptosis were present, while cell proliferation had recovered to sham-control levels at 5 weeks after asphyxia. It is important to note that we previously reported that numbers of caspase-3 and Ki-67-labeled cells were low even after 3 days recovery, when bulk cell death occurs.^12^ Thus, the anti-apoptotic effects of IGF-1 are unlikely to have contributed to improved white matter maturation in the present study. A limitation of the present study is that caspase-3 and Ki-67-positive cells were not assessed at time-points between 3 and 35 days after asphyxia. Thus, we cannot exclude the possibility that cell proliferation and apoptosis may have been transiently enhanced during IGF-1 infusion. This should be addressed in future studies.

In the present study, overt white matter atrophy was associated with ventriculomegaly at 3 days, and cystic white matter infarcts in four of eight asphyxia-vehicle foetuses after 5 weeks recovery. This is consistent with recent meta-analyses that identified perinatal HI as significant risk factor for cystic white matter injury.^69^ Although rates of severe white matter injury have fallen over time to ∼2–7% in high income countries, it remains highly associated with cerebral palsy in extremely preterm infants.^70^ In preterm infants, cystic white matter injury typically develops 3 to 5 weeks after birth, consistent with the present study.^63^ Cystic infarcts in our cohort were located adjacent to the lateral ventricles’ trigone, and at the level of frontal cerebral white matter near the foramen of Monro, consistent with reports in human preterm infants.^71,72^ Notably, this did not correlate with ventriculomegaly, although there was considerable variation. Nevertheless, the phenotype in the present study is consistent with cystic white matte injury of prematurity,^73^ with disintegration of cell elements, microglial aggregation and astroglial infiltration.^12,29^

Although the present study was not powered to determine whether IGF-1 could alleviate infarction, encouragingly only one of four IGF-1 treated foetuses in the present study developed cystic infarctions. Delayed neural inflammation after HI clears cell debris and helps support white matter repair and restoration.^60,74^ However, excessive priming of astrocytes and microglia has been associated with sustained secretion of pro-inflammation factors, including cytokines and ILs, and invasion of leukocytes into the brain parenchyma, which in turn are associated with white matter injury.^75^ Clinically, increased levels of circulating inflammation-related proteins after two weeks from birth is associated with reduced regional and total cerebral volumes, and IQ in childhood.^76^ It is unknown how IGF-1 facilitated long-lasting suppression of inflammation in this study. However, it is likely mediated by a combination of direct cell effects and indirect trophic effects.^11,77^

Finally, IGF-1 was associated with a long-term improvement of EEG power, but not spectral frequency, compared to the asphyxia-vehicle foetuses. In part, synchronous EEG activity reflects cortico-striatal and cortico-thalamo neural feedback, suggesting a restoration of subcortical neurons or axonal transmission in IGF-1-treated foetuses. Supporting this, in asphyxiated foetal sheep, reduced basal ganglia dysfunction after IGF-1 infusion was associated with preservation of striatal cholinergic and GABAergic neurons after 4 days.^78^ Further, MBP-myelin disruption after cerebral ischaemia in near-term foetal sheep was correlated with gross axonopathy that co-localized with dysmorphic astrocytosis.^79^ This should be explored in further long-term IGF-1- treatment studies.

An important consideration is that the aetiology of preterm neurodevelopmental impairment is still poorly understood. Both clinical and preclinical evidence suggests that contributing factors likely include neuroinflammation, HI, barotrauma and hyperoxia, alone or in combination.^7,8,80^ In the present study we examined the response to severe HI, but it will be important to test IGF-1 after other insults as well in the future. Encouragingly, in the present study IGF-1 reduced tertiary neuroinflammation suggesting potential for benefit in other neuroinflammatory settings. The asphyxia-IGF-1 group had higher PaO2 values than sham controls, albeit within the normal range. Given that the 35 day groups were not different in weight at post-mortem, or other biochemical or physiological values, or their cardiovascular response to asphyxia, it is unlikely that this difference affected outcomes. Other limitations of the present study include the smaller number of IGF-1 treated foetuses that completed the 35 day recovery protocol, particularly limiting power to assess the impact on cystic white matter injury, and that cells were quantified in one 20 × magnification field in the periventricular white matter. Future studies should further corroborate these exciting findings, assessing a larger cohort of foetuses and wider sampling of larger white matter tracts using stereological techniques. Further, we were unable to double-label apoptotic or proliferating cells with other glial markers due to species cross-over and the lack of cell specificity of markers tested for the ovine species.

## Conclusion and perspectives

The current findings support the concept that sustained, neural inflammation is a key mediator of slow evolving, white matter injury after HI. Improved white matter maturation, with normalisation of numbers of mature oligodendrocytes after very delayed treatment with the trophic factor IGF-1 suggests that surprisingly delayed therapeutic strategies may have the potential to improve neurodevelopmental outcomes in high-risk extremely preterm infants. Further studies are needed to determine the optimal therapeutic regimen, assess response to other causes of neuroinflammation and assess the viability of alternate routes of administration.

## Supplementary Material

fcae373_Supplementary_Data

## Data Availability

Data are available from the authors on reasonable request.
