# Supplementary material for: White matter protection with insulin-like growth factor-1 after hypoxia-ischaemia in preterm foetal sheep
Source: Brain Commun. 2024 Oct 24;6(6):fcae373. doi: 10.1093/braincomms/fcae373 (PMC11539755; doi:10.1093/braincomms/fcae373)
Supplement: fcae373_Supplementary_Data [file fcae373_supplementary_data.pdf]

Supplementary Materials

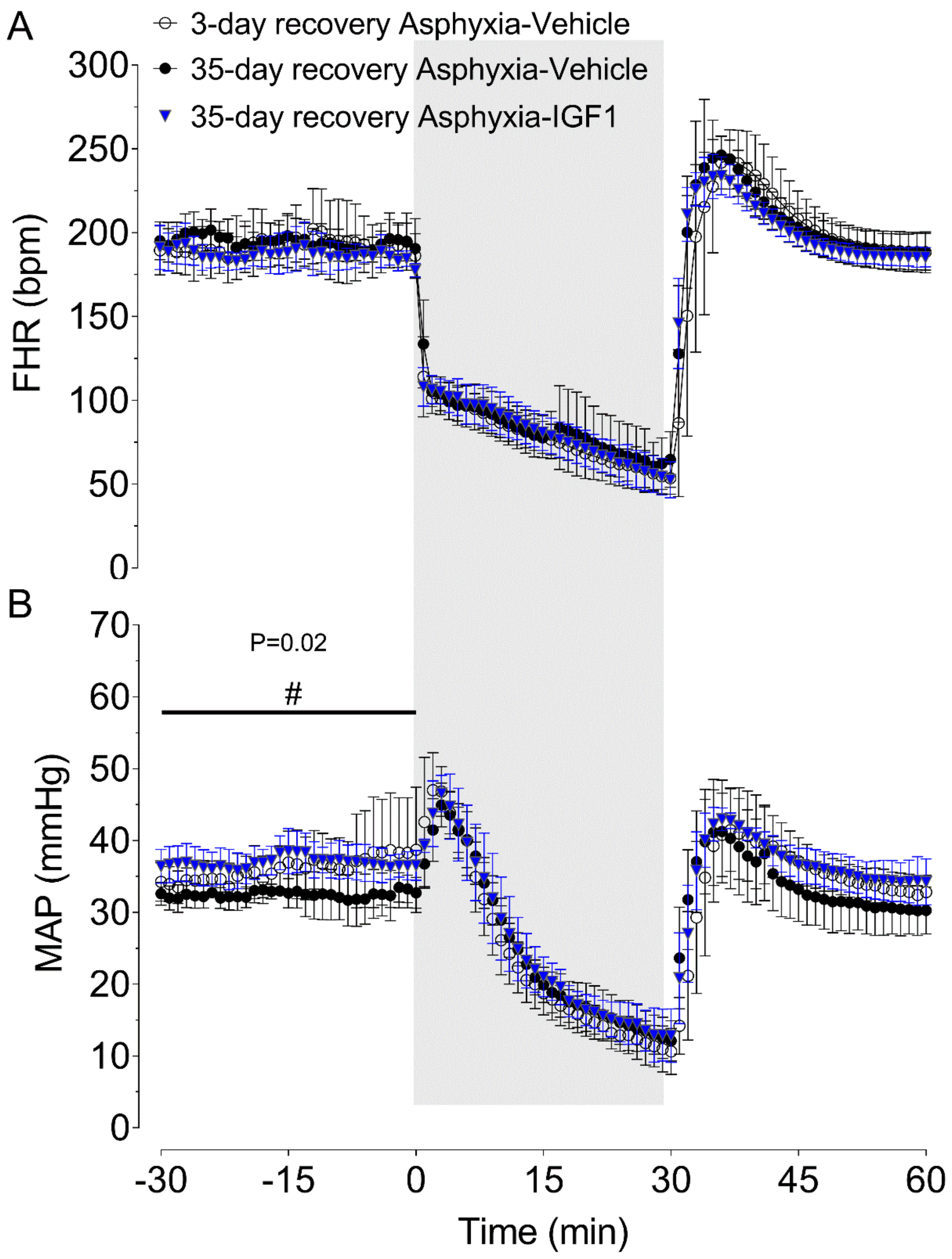

Supplementary Figure 1: Fetal heart rate (FHR, upper panel) and mean arterial blood pressure (MAP, lower panel) responses from 30 min before until 30 min after cord occlusion in the asphyxia 3-day and 35-day (white and black circles, n = 8 each), and asphyxia 35-day-IGF-1 (blue triangles, n = 4), groups. Data are presented as mean ± SD; 1-min averages. Group comparisons by one-way ANOVA and LSD test for baseline, and repeated measures ANOVA for occlusion and recovery. MAP was higher in IGF-1 animals during baseline. There were no significant differences in FHR or MAP between groups during occlusion or recovery. #$P<0.05$, asphyxia 35-day vs. asphyxia 35-day-IGF-1. bpm, beat per minute. IGF-1, insulin-like growth factor-1.

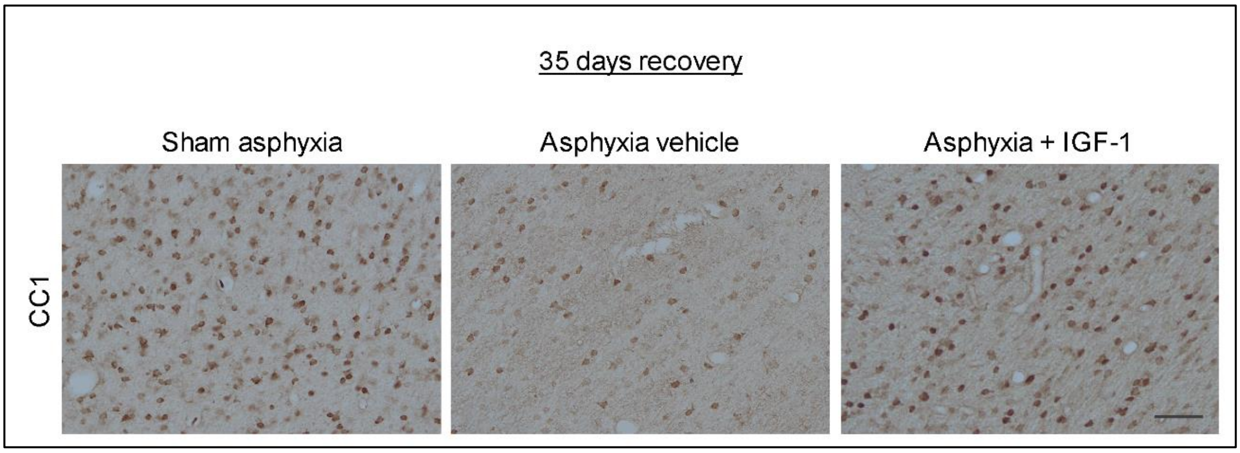

Supplementary Figure 2: Photomicrographic examples of mature oligodendrocytes. Mature CC1-positive oligodendrocytes in periventricular white matter of sham 35-days (left panel), asphyxia 35-days (middle panel), and asphyxia 35-days-IGF-1 animals (right panel). Scale bar = 50 µm. IGF-1, insulin-like growth factor-1. CC1, anti-adenomatous polyposis coli clone CC1.

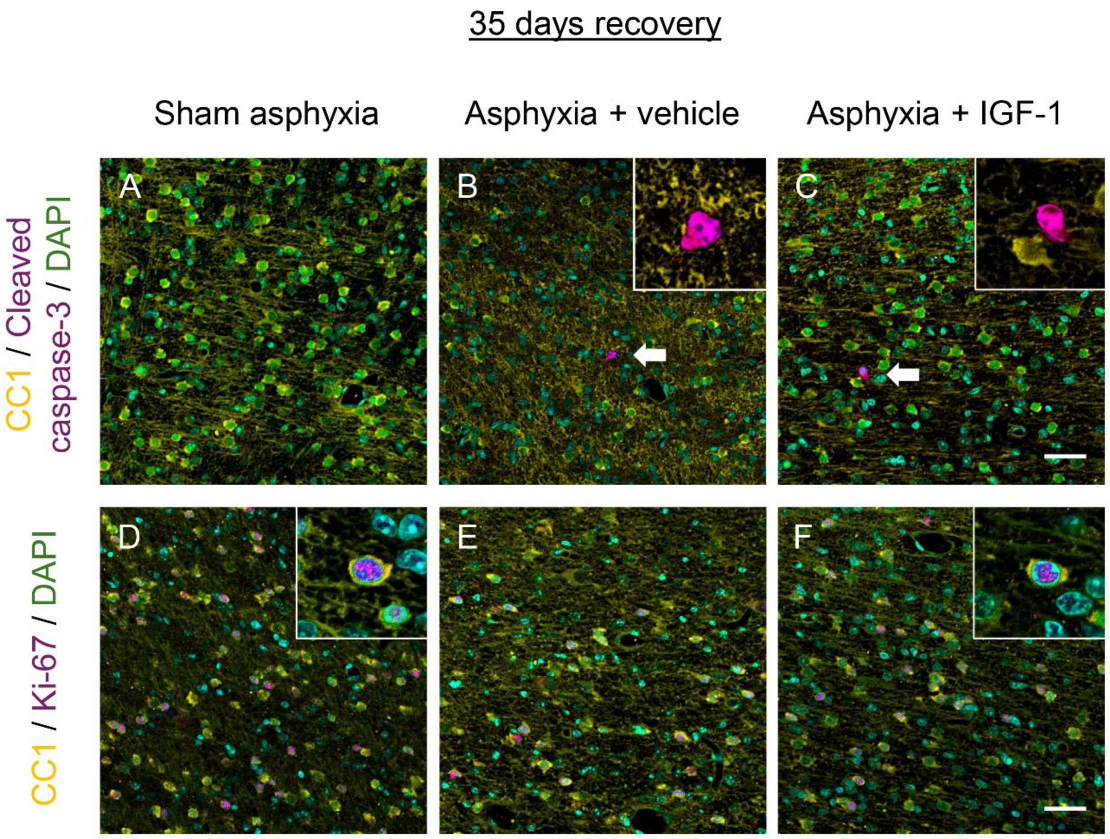

Supplementary Figure 3: Immunofluorescence. Co-localisation of mature oligodendrocytes (CC1) with cleaved caspase-3 (panels A-C) and Ki-67 (panels D-F) in the periventricular white matter of sham 35-days (Left), asphyxia 35-days (middle), and asphyxia 35-days-IGF-1 (right) animals. All images were taken at x20 magnification. Insets are x3 enlargements. Scale bar = 50 µm. IGF-1, insulin-like growth factor-1. CC1, anti-adenomatous polyposis coli clone CC1. DAPI, 4′,6-diamidino-2-phenylindole.
